# The Sensitivity of a Hexagonal Au Nanohole Array under Different Incident Angles

**DOI:** 10.3390/bios13060654

**Published:** 2023-06-15

**Authors:** Kang Yang, Meiying Li

**Affiliations:** School of Physics and Optoelectronic Engineering, Yangtze University, Jingzhou 434023, China; 2022720379@yangtzeu.edu.cn

**Keywords:** surface plasmon resonance, sensor, Au nanohole array, incident angle, sensitivity

## Abstract

Surface plasmon resonance sensors have been widely used in various fields for label-free and real-time detection of biochemical species due to their high sensitivity to the refractive index change of the surrounding environment. The common practices to achieve the improvement of sensitivity are to adjust the size and morphology of the sensor structure. This strategy is tedious and, to some extent, limits the applications of surface plasmon resonance sensors. Instead, the effect of the incident angle of excited light on the sensitivity of a hexagonal Au nanohole array sensor with a period of 630 nm and a hole diameter of 320 nm is theoretically investigated in this work. By exploring the peak shift of reflectance spectra of the sensor when facing a refractive index change in (1) the bulk environment and (2) the surface environment adjacent to the sensor, we can obtain the bulk sensitivity and surface sensitivity. The results show that the bulk sensitivity and surface sensitivity of the Au nanohole array sensor can be improved by 80% and 150%, respectively, by simply increasing the incident angle from 0° to 40°. The two sensitivities both remain nearly unchanged when the incident angle further changes from 40° to 50°. This work provides new understanding of the performance improvement and advanced sensing applications of surface plasmon resonance sensors.

## 1. Introduction

Surface plasmon resonance (SPR) supported by noble metal nanostructures can concentrate optical fields into nanoscale space and significantly enhance the near-field intensity [1,2,3], bringing in sensitive response to the refractive index change of the surrounding environment [4,5,6,7]. Because of this characteristic, SPR sensors have been widely used in the fields of medical diagnosis, food safety regulation and environmental monitoring in the past few decades [8,9,10,11,12,13,14,15,16]. The sensing performance of an SPR sensor can be evaluated by sensitivity, which is defined as the spectral shift of the sensor per refractive index unit (RIU) [17]. The pursuit of high sensitivity has therefore attracted much attention in the field of surface plasmon resonance sensing.

The traditional methods to obtain high sensitivity mainly focus on the construction of SPR sensors with strong near-field enhancement. Some sensor nanostructures, such as nanohole [18,19,20,21], nanoring [22,23,24,25], nanodisk [26], nanodimer [27] and closely packed nanoclusters [28] with narrow nanogap, have been demonstrated to be highly sensitive to slight changes in the surrounding medium. However, the strategy relies heavily on the size/gap of the SPR sensor structures. Another effective way to improve sensitivity is to couple different resonance modes into an SPR sensor structure to greatly enhance the near-field intensity [29,30,31,32]. For example, Cetin and Altug fabricated an asymmetric ring/disk structure on a Au layer to produce Fano resonance [33]. Compared with the concentric ring/disk structure, the asymmetric structure exhibited a noticeably stronger near-field intensity, resulting in a higher sensitivity. As another example, Ye’s group proposed a Au nanoring-SiO_2_ spacer-Au film nanostructure [34]. Compared with the Au cavity structure made of two Au films, the sandwich configuration can generate a strong Fano resonance by coupling the localized surface plasmon resonance (LSPR) mode of the nanoring array and the cavity mode of the structure. Therefore, the near-field distributions can be transferred from the SiO_2_ layer to the sensor surface, which allows the access of electromagnetic sites by the surrounding target. In addition, the near-field intensity can be significantly enhanced, leading to an excellent sensing performance. The challenge of this strategy is that it requires an elaborate structure design to achieve the effective couplings of different resonance modes. In addition to the structural size and morphology, material composition can also influence the sensitivity of an SPR sensor. Maier et al. investigated and compared the refractive index sensing sensitivity of 17 materials, including noble metals, refractory metals, transition metal nitrides and conductive oxides [35]. Ag shows the highest sensitivity due to its extremely low plasmon loss compared with other materials. However, Ag is easily oxidized under ambient conditions; Au is popularly chosen due to its excellent chemical stability and excellent plasmonic property [36,37].

Alternatively, the sensitivity of an SPR sensor may be improved by adjusting the incident angle of excited light. In the work of Odom’s group, they showed that the average near-field intensity of a two-dimensional Au nanoparticle array can be improved noticeably as the incident angle increases [38]. It means that the adjustment of incident angle could be a convenient way to tune the sensitivity of an SPR sensor. In this work, we utilized a 3D finite difference time-domain (FDTD) method and theoretically investigated the effect of incident angle on the sensitivity of a widely used hexagonal Au nanohole array sensor [17,20]. Interestingly, we found that the bulk sensitivity and the surface sensitivity in different spatial regions away from the sensor surface can be significantly improved by increasing the incident angle from 0° to 40°. The sensitivity improvement can be negligible as incident angle further increases from 40° to 50°. Compared with the common strategy focusing on the morphology/size/composition adjustment, this work provides a simple way for the sensitivity improvement of SPR sensors.

## 2. Methods

The simulation method is similar to our previous work [17]. Briefly, the FDTD simulation (Lumerical Solutions, Vancouver, Canada) was performed to investigate the resonance properties of the hexagonal Au nanohole array (the array period was 630 nm, the hole diameter was 320 nm and the array thickness was 100 nm), where the periodic boundary conditions in the xy-plane (structured surface plane) and perfectly matched layer conditions at the z axis were adopted. The simulation time was set to 1000 fs to guarantee the convergence. The Yee cell size was 2 nm × 2 nm × 2 nm. The dielectric functions of Au and Si were taken from a multi-coefficient fitting model offered by the FDTD software (2020 R2).

Figure S1 (Supplementary Materials) gives the flow chart of the manufacturing process of the hexagonal Au nanohole array. Firstly, the lift-off and SU-8 2000.5 resists were coated on a silicon wafer by spin coating. The substrates then underwent holographic lithography, where three prefabricated diffractive gratings orientated 120° to each other produced an ordered periodic pattern on the photoresist-coated substrate because of the interference of the first-order laser beams from the gratings. Then, reactive ion etching (RIE) was used to remove the exposed lift-off resist with the SU-8 2000.5 nanopillar array as the mask. Afterwards, a Au film was deposited on the substrate by electron beam evaporation. Thus, the hexagonal Au nanohole array was finally obtained after the lift-off process.

## 3. Results and Discussion

**Figure 1:**
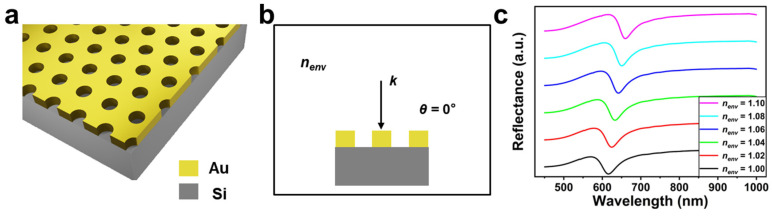
(**a**) The schematic and (**b**) excitation view of the hexagonal Au nanohole array sensor. (**c**) The reflectance spectra of the hexagonal Au nanohole array sensor when $n_{env}$ increases from 1.00 to 1.10 with a step of 0.02. *θ* is 0°.

Figure 1a illustrates the schematic view of the hexagonal Au nanohole array sensor. The period of the array sensor (the spacing between the center of two adjacent nanoholes) was 630 nm. The diameter and height of the nanohole were 320 nm and 100 nm, respectively. We first simulated the spectral response of the array sensor to refractive index change of the surrounding environment ($n_{env}$) when the incident angle (*θ*) was 0° (namely, under a normal incidence condition, see Figure 1b). Figure 1c shows the obtained reflectance spectra of the Au nanohole array when $n_{env}$ was of 1.00 to 1.10 with a step of 0.02. It is clear that the reflectance spectrum continuously shifts to a longer wavelength with an increase in $n_{env}$, demonstrating that the Au nanohole array is sensitive to the change of the surrounding medium.

**Figure 2:**
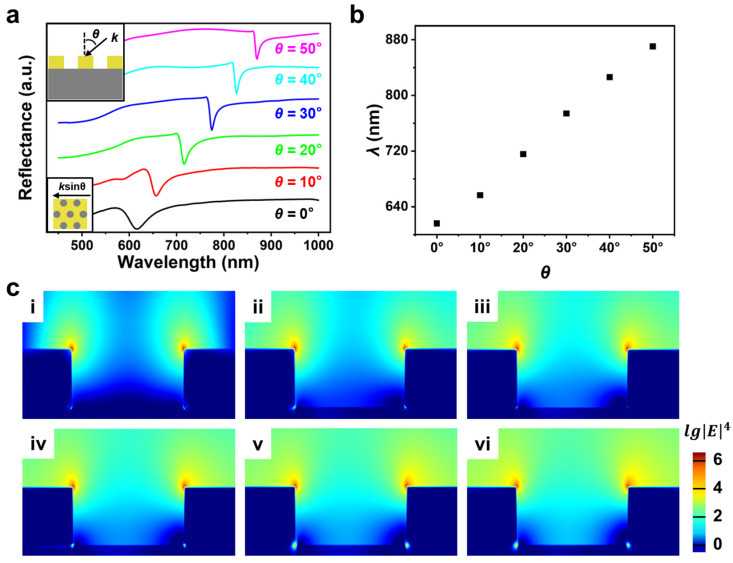
(**a**) The reflectance spectra of the hexagonal Au nanohole array sensor when *θ* (shown in the inset) increases from 0° to 50° with a step of 10°. (**b**) The peak positions (*λ*) of the Au nanohole array sensor under different *θ*. (**c**) The near-field distributions of the Au nanohole array when *θ* is 0° (**i**), 10° (**ii**), 20° (**iii**), 30° (**iv**), 40° (**v**) and 50° (**vi**). $n_{env}$ is 1.00.

Next, we investigated the resonance property of the Au nanohole array under oblique incidence. The corresponding excitation view can be found in the inset of Figure 2a, where *θ* ranges from 0° to 50° with a step of 10° and $n_{env}$ is 1.00. From the results shown in Figure 2a, we can see the reflectance spectrum red shifts noticeably as *θ* increases. Figure 2b displays the peak position (*λ*) of the nanohole array under different *θ*. It is clear that *λ* increases from about 620 nm to about 870 nm as *θ* changes from 0° to 50°. Since sensing sensitivity mainly depends on the electromagnetic field intensity of SPR sensors, we further analyzed the near-field distributions of the Au nanohole array under different *θ*, as exhibited in Figure 2c. On the one hand, the near-field distributions of the array sensor are mainly localized at the top edge of the Au nanohole. On the other hand, we can see that the enhanced electromagnetic sites can be also observed at the bottom edge of the nanohole when *θ* is within a large range (such as 40°; see Figure 2c(v)). In addition, it is clear that the near-field intensity of the Au nanohole array is improved from about 10^5^ to 10^6^ (see the top edge of the nanohole) as *θ* increases from 0° to 50°. This indicates that a better near-field enhancement can be obtained by increasing *θ* and thus improve the sensitivity of the Au nanohole array sensor.

**Figure 3:**
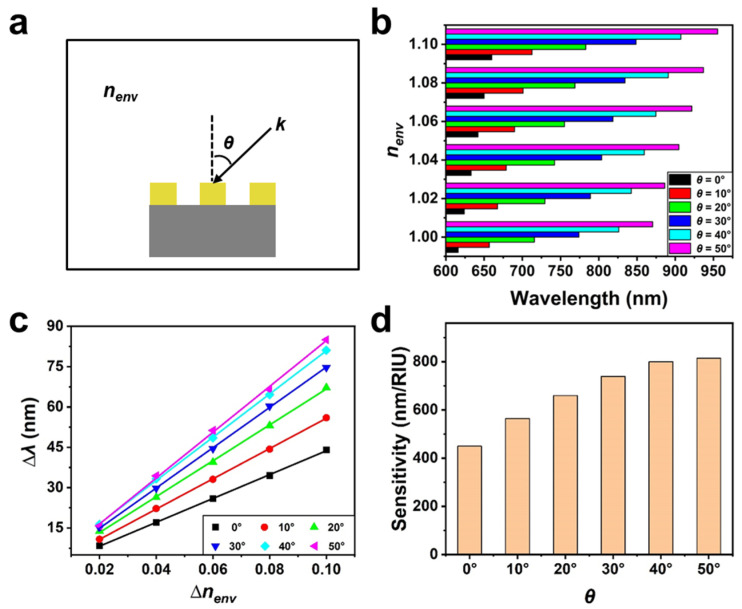
(**a**) The excitation diagram of the hexagonal Au nanohole array sensor. (**b**) The peak positions of the Au nanohole array sensor under different $n_{env}$ and *θ*. (**c**) The linear relationship between ∆*λ* and $∆n_{env}$. (**d**) The obtained bulk sensitivities of the Au nanohole array sensor under different *θ*.

We then focused on the difference in the sensing sensitivity of the Au nanohole array sensor when *θ* ranged from 0° to 50°. Figure S2 (Supplementary Materials) shows the obtained reflectance spectra of the array sensor under different *θ* and $n_{env}$ conditions (The excitation view is displayed in Figure 3a). We can see that the resonance spectrum shifts toward longer wavelength noticeably when $n_{env}$ rises from 1.00 to 1.10, regardless of *θ*. Figure 3b,c illustrate *λ* and the corresponding spectral shift (∆*λ*, compared with the inherent *λ* when $n_{env}$ is 1.00) of the nanohole array, respectively. According to sensitivity = ∆*λ*/$∆n_{env}$, we can obtain the bulk sensitivity (shown in Figure 3d) of the array sensor under different *θ* by calculating the slope of each curve (see Figure 3c). It can be seen that the sensitivity of the Au nanohole array sensor rises from about 450 nm/RIU to about 800 nm/RIU as *θ* increases from 0° to 40° and remains nearly unchanged when *θ* further increases from 40° (800 nm/RIU) to 50° (815 nm/RIU). The phenomenon is consistent with the variation trend of near-field intensities shown in Figure 2c, demonstrating the feasibility of improving the sensitivity of the Au nanohole array sensor by increasing *θ* appropriately.

**Figure 4:**
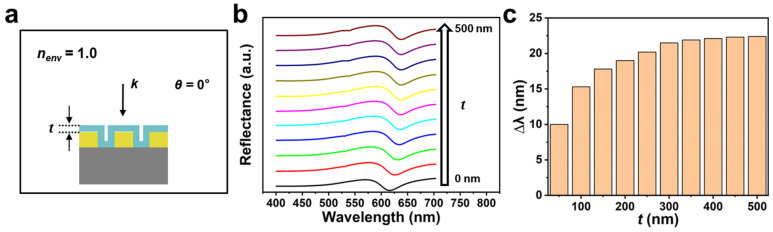
(**a**) The schematic view of the hexagonal Au nanohole array sensor covered by a layer of target (n = 1.05) with a thickness of *t*. (**b**) The reflectance spectra and (**c**) ∆*λ* of the Au nanohole array sensor when *t* ranges from 0 nm to 500 nm with a step of 50 nm. $n_{env}$ = 1.00 and *θ* is 0°.

In the previous section, we analyzed the change in sensitivity of the Au nanohole array sensor under different *θ* when facing a refractive index variation in the bulk environment. We then focused on the surface sensitivity of the Au nanohole array in different spatial regions away from the sensor surface. To facilitate the subsequent analysis, a target layer of n = 1.05 was applied to the surface of the nanohole array when $n_{env}$ was 1.00, as shown in Figure 4a. Figure 4b exhibits the change in the reflectance spectra (*θ* = 0°) of the Au nanohole array when the thickness (*t*) of the target ranged from 0 nm to 500 nm with a step of 50 nm. We can see that the peak position of the Au nanohole array at first noticeably shifts towards longer wavelengths as *t* increases, but the moving speed slows down gradually. The reflectance spectrum keeps nearly the same when *t* is large enough (in the range from 300 nm to 500 nm). Figure 4c shows ∆*λ* (compared with the inherent *λ* of the Au nanohole array when *t* is 0 nm and $n_{env}$ is 1.00) of the Au nanohole array sensor under different *t*. It is clear that ∆*λ* increases from about 10 nm to about 22 nm when *t* changes from 50 nm to 300 nm and remains nearly unchanged when *t* further increases from 300 nm to 500 nm.

**Figure 5:**
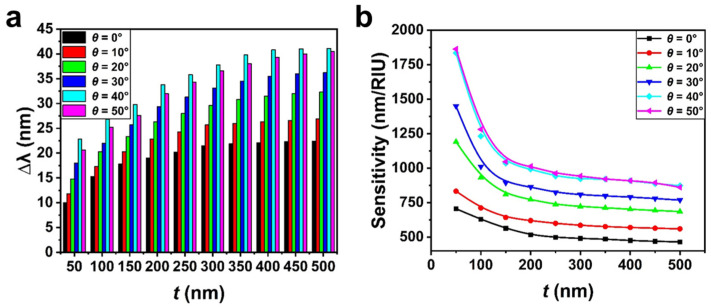
(**a**) The obtained ∆*λ* and (**b**) surface sensitivities of the hexagonal Au nanohole array when *t* ranges from 50 nm to 500 nm with a step of 50 nm and *θ* ranges from 0° to 50° with a step of 10°. $n_{env}$ = 1.00.

In addition to the normal incidence condition, we also obtained the reflectance spectra change of the Au nanohole array sensor at different *t* when *θ* ranged from 0° to 50° with a step of 10°, as shown in Figure S3 (Supplementary Materials). It can be seen that the resonance spectrum of the array sensor red shifts significantly with the increase in *t* when *t* is small (such as in the range of 0 nm to 200 nm), but remains nearly unchanged when *t* falls within a much higher range (such as from 400 nm to 500 nm), regardless of *θ*. The change in ∆*λ* with *t* under each *θ* and the corresponding comparison results are illustrated in Figure S4 (Supplementary Materials) and Figure 5a, respectively. Under a fixed *t*, it is clear that ∆*λ* increases noticeably with an increase in *θ*. For example, when *t* is 300 nm, ∆*λ* values reach about 21 nm, 25 nm, 30 nm, 33 nm, 38 nm and 37 nm when *θ* is 0°, 10°, 20°, 30°, 40° and 50°, respectively. This indicates that the response performance of the Au nanohole array sensor to slight changes in refractive index within different spatial regions away from the sensor surface can be tuned by adjusting *θ*. The quantitative analysis of the surface sensitivity of the Au nanohole array sensor is different from the calculation method of bulk sensitivity shown in Figure 3c and can be approximately achieved through the following equation [39,40]:

(1) $$m=\frac{∆\lambda }{\left(n_{adsorbate}-n_{env}\right)\left(1-e^{\frac{-2t}{l_{d}}}\right)}$$

where m (nm/RIU) is the sensitivity factor (namely sensing sensitivity), $n_{adsorbate}$ and $n_{env}$ are the refractive index values of the target ($n_{tar}$ = 1.05) and the environment ($n_{env}$ = 1.00), respectively, and $l_{d}$ is the decay length of the near field of the Au nanohole array sensor. Under normal incidence, $l_{d}$ is set as 300 nm for the calculation, considering ∆*λ* nearly remains unchanged when *t* reaches 300 nm (see Figure S4a, Supplementary Materials). Similarly, we chose $l_{d}$ as 350 nm when *θ* ranged from 10° to 40° and as 400 nm when *θ* was 50° (Figure S4b–f, Supplementary Materials). On the one hand, the setting is approximately consistent with the calculation results of the change in ∆*λ* at different *t* when *θ* increases from 0° to 50°. On the other hand, we can see the variation of $l_{d}$ under different *θ* according to the formula [41] $l_{d}=\frac{\lambda }{2\pi }{\left|\frac{\varepsilon_{d}+\varepsilon_{m}^{’}}{\varepsilon_{d}^{2}}\right|}^{\frac{1}{2}}$, where λ represents resonance wavelength, $\varepsilon_{d}$ and $\varepsilon_{m}^{’}$ are dielectric constants of the surrounding environment and metal material (real part), respectively. When *θ* increases from 0° to 50°, λ of the Au nanohole array sensor shows significant red shift (displayed in Figure 2a,b). In this case, a larger $l_{d}$ can be achieved, roughly consistent with the above setting. Figure 5b shows the calculated surface sensitivities of the Au nanohole array sensor under different *t* and *θ* conditions. When *θ* is fixed (such as 20°), it is clear that as *t* increases from 50 nm to 500 nm, the obtained sensitivity value decreases dramatically at first and then remains nearly unchanged. This indicates that the response sensitivity of the Au nanohole array weakens significantly as the spatial region where the refractive index varies moves away from the sensor surface. This can be attributed to the rapid decay in near-field intensity with increased distance away from the surface of the Au nanohole array sensor. When *t* is large enough (such as 500 nm), we can see that the obtained surface sensitivities under each *θ* are almost equal to the corresponding bulk sensitivities shown in Figure 3d. This illustrates that the target layer completely serves as the bulk medium background of the Au nanohole array in this case. In addition, the phenomenon demonstrates the feasibility of the setting values of $l_{d}$ used in Equation (1) for the calculation of surface sensitivities under different *θ*. When only the change in *θ* is considered, we can see the surface sensitivity of the Au nanohole array sensor rises significantly with the increase in *θ* from 0° to 40° and remains nearly unchanged when *θ* further increases from 40° to 50°. For example, at a *t* of 50 nm, the obtained surface sensitivity rises from about 750 nm/RIU to 1850 nm/RIU when *θ* increases from 0° to 40°/50°, nearly a 1.5 times improvement. The sensitivity changes of the Au nanohole array sensor under different *θ* when facing a slight variation in the refractive index of the bulk environment (shown in Figure 3d) and in different spatial regions away from the sensor surface (shown in Figure 5b) together demonstrate the possibility of tunning sensing performance of the hexagonal Au nanohole array by adjusting *θ*.

## 4. Conclusions

In summary, with the utilization of the FDTD method, we have systematically studied the effect of incident angle on the sensing sensitivity of a hexagonal Au nanohole array sensor. By adjusting the incidence angle from 0° to 40°, the bulk sensitivity of the sensor can be increased gradually and eventually reaches an 80% improvement. Meanwhile, the surface sensitivity can be improved by 1.5 times. Both the bulk sensitivity and surface sensitivity remain nearly unchanged with the further change in incidence angle from 40° to 50°. The incident angle-dependent sensitivity shown in this work can be used to optimize the sensing performance of SPR array sensors, which provides new understanding for advanced sensing applications of SPR sensors in the future.

## Data Availability

Data will be available from the author on request.

## Supplementary Materials

The following supporting information can be downloaded at: https://www.mdpi.com/article/10.3390/bios13060654/s1, Figure S1: Preparation procedure of the Au nanohole array. Figure S2: The reflectance spectra of the Au nanohole array when $n_{env}$ increases from 1.00 to 1.10 with a step of 0.02. Figure S3: The reflectance spectra of the Au nanohole array when *t* increases from 0 nm to 500 nm with a step of 50 nm. Figure S4: ∆*λ* of the Au nanohole array when *t* increases from 0 nm to 500 nm with a step of 50 nm.
