# Supplementary material for: The Sensitivity of a Hexagonal Au Nanohole Array under Different Incident Angles
Source: Biosensors (Basel). 2023 Jun 15;13(6):654. doi: 10.3390/bios13060654 (PMC10296690; doi:10.3390/bios13060654)
Supplement: Supplementary file 1 [file biosensors-13-00654-s001.zip › biosensors-2448730-supplementary.pdf]

biosensors MDPI

Supplementary Materials

The Sensitivity Research of a Hexagonal Au Nanohole Array Under Different Incident Angle

Kang Yang* and Meiying Li

School of Physics and Optoelectronic Engineering, Yangtze University, Jingzhou 434023, China.

*Email address: kangyang@yangtzeu.edu.cn

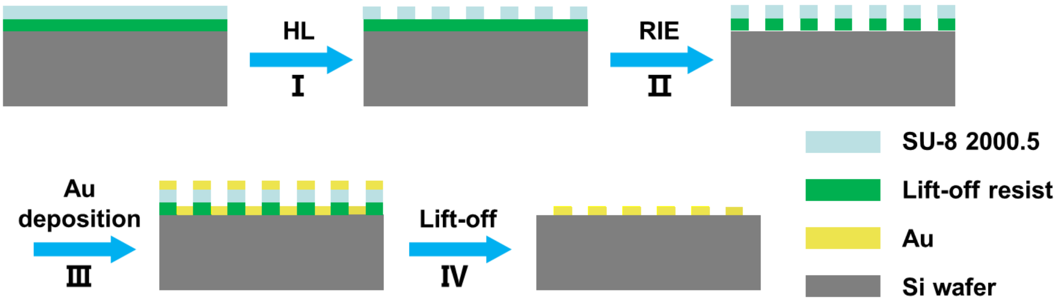

Figure S1. Preparation procedure of the Au nanohole array. (I) Holographic lithography (HL). (II) Reactive-ion etching (RIE). (III) Au deposition. (IV) Lift-off process.

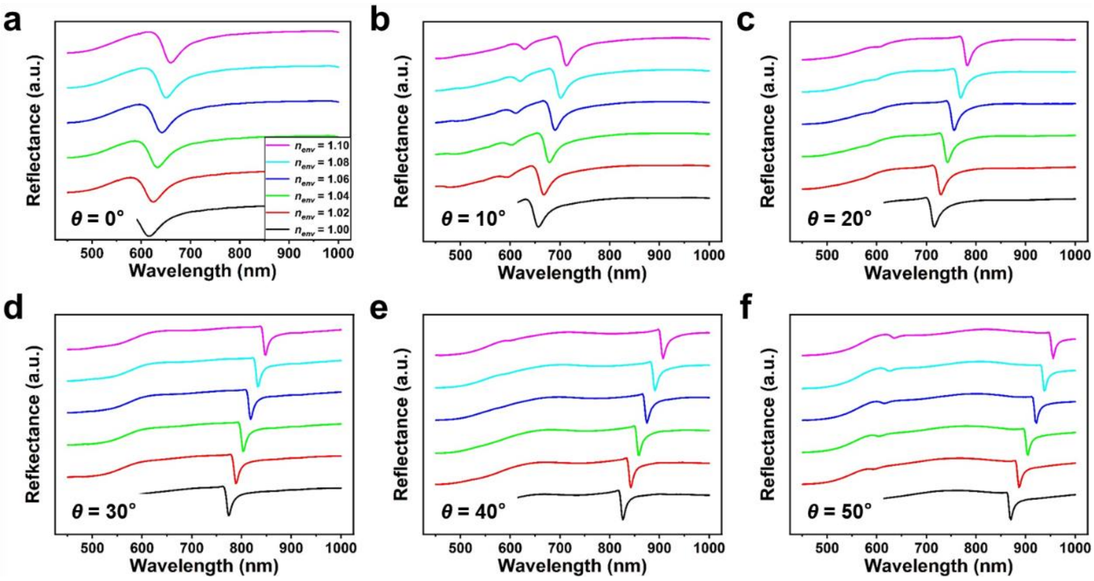

Figure S2. The reflectance spectra of the Au nanohole array when n_{env} increases from 1.00 to 1.10 with a step of 0.02. From (a) to (f), θ is 0°, 10°, 20°, 30°, 40° and 50°, respectively.

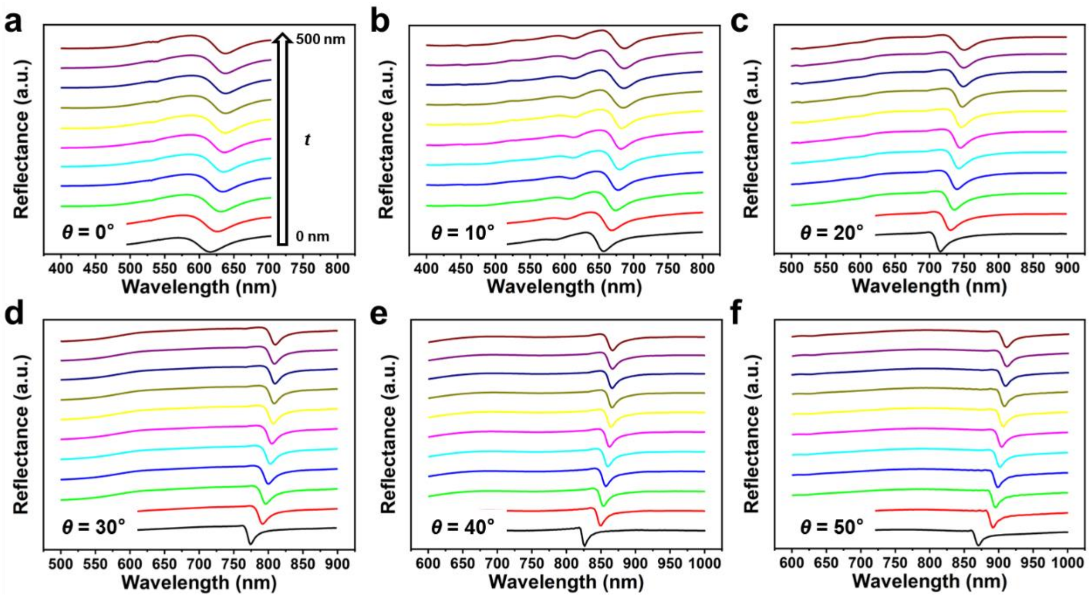

Figure S3. The reflectance spectra of the Au nanohole array when *t* increases from 0 nm to 500 nm with a step of 50 nm. From (a) to (f), θ is 0°, 10°, 20°, 30°, 40° and 50°, respectively.

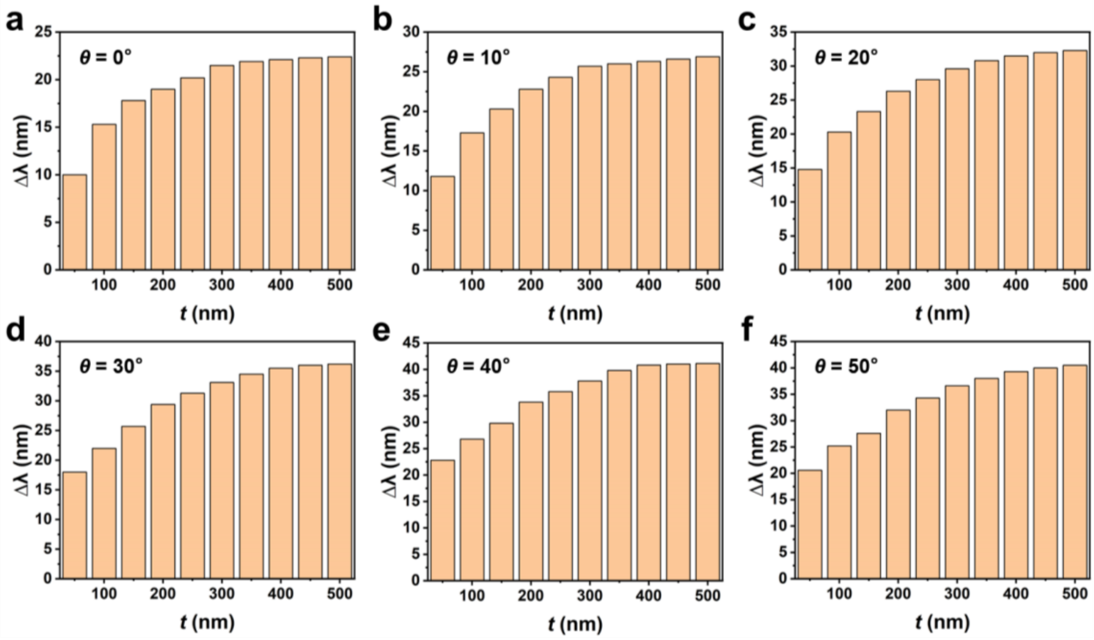

Figure S4. $\Delta\lambda$ of the Au nanohole array when t increases from 0 nm to 500 nm with a step of 50 nm. From (a) to (f), θ is 0°, 10°, 20°, 30°, 40° and 50°, respectively. n_{env}=1.00.
